# Lowering Red Meat and Processed Meat Consumption With Environmental, Animal Welfare, and Health Arguments in Italy: An Online Experiment

**DOI:** 10.3389/fpsyg.2022.877911

**Published:** 2022-05-18

**Authors:** Arie Dijkstra, Valentina Rotelli

**Affiliations:** ^1^Department of Social Psychology, University of Groningen, Groningen, Netherlands; ^2^Department of Psychology, Catholic University of the Sacred Heart, Milan, Italy

**Keywords:** meat consumption, persuasion, environment, animal welfare, health

## Abstract

**Introduction:**

In addition to being a source of valuable nutrients, meat consumption has several negative consequences; for the environment, for animal welfare, and for human health. To persuade people to lower their meat consumption, it is assumed that the personal relevance of the topic of lowering meat consumption is important as it determines how people perceive the quality of the arguments.

**Method:**

In an experimental exploratory field study (*n* = 139), participants recruited from the general Italian population were randomized to one of the four conditions with a text with pictures on the environmental, animal welfare, or health consequences of meat consumption, or a text on mustard (the control condition). The dependent variables were self-reported consumption of red meat and processed meat after 2 weeks. Personal relevance was assessed in the pre-test with self-reported meat consumption and intention.

**Results:**

The interaction between pre-test meat consumption and condition was significant: In participants who scored high on pre-test meat consumption, the self-reported red meat consumption after 2 weeks in the health argument condition was significantly lower compared to the control condition and the environmental argument condition. The effects of pre-test intention as a moderator were less certain.

**Discussion:**

The persuasive effects of the different arguments made a difference only in people who ate a relatively high level of meat in pre-test, and the type of arguments made a difference. Although the present outcomes are caused by the specific formulations of the arguments in this study, the results do show that it is relevant to choose the arguments carefully to ensure effectiveness.

## Introduction

Meat consumption nowadays is not only perceived as a source of valuable proteins to maintain the human physical body and related hedonistic experience of satisfying a human need, but also as a source of various direct and indirect negative consequences (Godfray et al., 2018). That is, large-scale meat production has indirect negative consequences for the environment and for animal welfare, while meat consumption itself can have direct negative physical consequences. The urgency of these effects is also reflected in the emerging scientific field (Moreira et al., 2022).

Lowering meat consumption is one important angle to avoid these indirect and direct negative consequences. One important strategy to lower meat consumption is to provide individuals with information about the relationship between meat consumption and various negative consequences, in well-designed persuasive messages. There are at least three core arguments that might be used in persuasive messages: environmental consequences, animal welfare consequences, and health consequences. To start with, an ultra-brief summary of some evidence of these consequences of meat consumption and the acceptance of such information in the population is presented below.

### Environmental Consequences

The current meat production sector contributes to several aspects of environmental problems (Garnett, 2009; Herrero et al., 2015; Macdiarmid et al., 2016). According to the Starke (2009), the livestock sector accounts for 51% of global greenhouse gas emissions and is responsible for methane production (de Boer and Aiking, 2011; Westhoek et al., 2011; Graham and Abrahamse, 2017). The meat industry occupies 83% of agricultural land, consumes 70% of all cereals produced worldwide (FAO, 2006), and it consumes large quantities of drinking water and pollutes drinking water (Hooda et al., 2000; Audsley et al., 2010; Clonan et al., 2015).

Despite the available data on the factual environmental consequences of meat production, many people still seem unaware of the effects of their meat consumption on the environment (Macdiarmid et al., 2016; Camilleri et al., 2019; Bschaden et al., 2020). Recent reviews conclude that most consumers are not ready to make food choices based on environmental arguments (Austgulen et al., 2018; Sanchez-Sabate et al., 2019).

### Animal Welfare Consequences

Regarding animal welfare, research suggests that animals are sentient beings, capable of experiencing emotions such as fear, anxiety, and suffering (Désiré et al., 2002). Leading a life in captivity is stressful for animals, and is associated with self-harm, cannibalism, and aggression. For this reason. animals usually undergo various mutilations to make them harmless. For instance, their beak is cut, their tail is amputated, the horns are removed, or their teeth are broken (Nuffield Council on Bioethics, 2005).

Regarding animal welfare, one study showed that 88.5% of the sample believed that it was important that the meat they buy has been produced with good animal welfare (Clonan et al., 2015), and some evidence has shown that the claim of animal-friendly production is associated with a higher product quality perception (Phan-Huy and Fawaz, 2003; Frewer et al., 2005). People view industrial production negatively and appreciate traditional and smaller farms, perceiving them with benefits such as more sanitary conditions, efficiency, and improved welfare (Bennett and Blaney, 2002; McKendree et al., 2014; Clark et al., 2016). According to many studies, the dominant motivational factor for being vegetarian is animal welfare (Beardsworth and Keil, 1991; Lindeman and Väänänen, 2000; Bastian et al., 2012; Stoll-Kleemann and Schmidt, 2017). The structure of the motivation to reduce meat consumption to improve animal welfare is complex, but it may be targeted in different ways to lower meat consumption (de Boer and Aiking, 2022a).

### Health Consequences

In terms of human health, many studies have highlighted the relationship between the consumption of red meat and processed meat with various diseases (Friel et al., 2009; McEvoy et al., 2012), including coronary heart disease, stroke, and diabetes (Tappel, 2007; Micha et al., 2010). Further, the World Health Organization, has classified red meat as probably carcinogenic to humans, and it has asserted that processed meat is carcinogenic (McGuire, 2016; also see Bouvard et al., 2015). However, other interpretations of the complex data are possible: a recent review concludes that current meat consumption does not need to change (Johnston et al., 2019).

Health concerns tend to rate relatively high compared to other potential motives although there are differences between sociodemographic groups (Wezemael et al., 2010; Tobler et al., 2011; Cordts et al., 2014). However, the idea of meat being essential for maintaining health and of vegetarian diets being nutritionally unbalanced still seems to be rather entrenched. These beliefs represent a barrier in reducing meat consumption, particularly among middle-aged people (Barr and Chapman, 2002; Graça et al., 2015).

### Persuasion

Lowering meat consumption might prevent these negative outcomes. For example, a modeling study based on Italian epidemiological data showed that reductions in meat consumption were associated with significant desired effects on life expectancy and CO_2_ emission (Farchi et al., 2017). One way to influence people to eat less meat is to persuade them with arguments (Bonnet et al., 2020; Harguess et al., 2020): persuasive messages might be designed, which link the three types of negative outcomes to individual meat consumption. However, from a theoretical point of view and verified in practice, it can be expected that people react differently to different arguments (Verain et al., 2017). That is, individual differences, present at the moment of exposure to the persuasive message may determine how the message is received and, subsequently, what the persuasive effects are.

In the field of health persuasion, personal relevance of the issue of persuasion has been consistently shown to influence persuasion: personal relevance is a predictor of defensive reactions toward the persuasive information that inhibit persuasion. In the line of research on the self-affirmation procedure in persuasion, defensiveness was found in participants who drank more alcohol (Harris and Napper, 2005), who consumed more caffeine (Reed and Aspinwall, 1998; Sherman et al., 2000), who were heavier smokers (Harris et al., 2007), and who were at high risk for diabetes (van Koningsbruggen and Das, 2009). These studies suggest that high objective personal relevance is associated with defensive reactions toward the persuasive information. Based on this assumption, in the present study it is expected that people who consume higher levels of meat can react more defensively toward persuasive messages. However, this reaction may occur especially with regard to certain arguments to lower meat consumption.

These effects of persuasive messages can be understood through the Elaboration Likelihood Model (ELM; Petty and Cacioppo, 1986; for an application to meat consumption persuasion see Weingarten et al., 2022). The ELM forwards that personal relevance is an important determinant of central processing of persuasive information; it leads to positive, neutral, or negative thoughts in reaction to the persuasive information. In central processing, persuasive arguments are carefully scrutinized and understood through the activation of long-term memory contents that manifest as thoughts. Thus, the abovementioned findings regarding high personal relevance predicting defensive reactions imply negative thoughts in reaction to the persuasive information (Cacioppo et al., 1997). Another central tenet of the ELM may now be relevant: The ELM proposes that when recipients process the information centrally—implying that they scrutinize the arguments carefully— they will notice the difference between so-called weak and strong arguments (Petty and Cacioppo, 1986; Park et al., 2007; Dijkstra and Ballast, 2012).

Strong arguments refer to concrete, direct, plausible, and undeniable outcomes that lead to vivid mental images that are experienced as relevant to oneself; they cannot be easily rejected with counter-arguments. Weak arguments refer to more indirect, probabilistic outcomes, brought about in a complex causal process that leads to more abstract and less vivid mental images that may seem less relevant to oneself. They can be rejected more easily and may seem “far-fetched,” and various counter-arguments may be applicable (Dijkstra and Ballast, 2012). Thus, argument quality is defined here on the basis of argument characteristics, and not solely on the basis of people's reactions to the arguments (Petty and Wegener, 1991; Dijkstra and Ballast, 2012).

The typical reactions toward weak arguments are negative thoughts, at least, in people for whom the topic is of personal relevance (Petty and Cacioppo, 1986; Dijkstra and Ballast, 2012). These negative thoughts are manifestations of defensive self-regulatory reactions that lower persuasion to cope with threat-related emotions (Dijkstra and Elbert, 2019). Also, in reaction to a persuasive message advocating lower meat consumption such defensive reactions have been traced (Piazza et al., 2015), also in psychophysiology (Spelt et al., 2019). This reactive phenomenon has also been conceptualized as meat-related cognitive dissonance, in which a perceived inconsistency in thinking leads to aversive arousal that activates “inconsistency-reduction strategies” (Rothgerber and Rosenfeld, 2021), in the present study referred to as defensive self-regulatory reactions.

In the present study of the three types of core arguments to lower meat consumption, we argue that environmental arguments will be perceived as relatively weaker, while health arguments will be perceived as relatively stronger. Environmental arguments might be seen as weaker because the outcomes are indirectly and collectively caused. This causal structure implies a shared responsibility that may be easily waived by individuals (Piazza et al., 2015). Indeed, in a qualitative study on lowering meat consumption participants were particularly skeptical about the effects of reducing meat consumption on the environment; many were unconvinced (Collier et al., 2021).

Health arguments, on the other hand, refer to direct outcomes under one's control and responsibility. These arguments may elicit fewer negative thoughts because they are less easy to deny. Moreover, the studies cited above on defensiveness in the domain of health persuasion suggest that those for whom the persuasive message is most relevant (here consuming a high level of meat) will especially get defensive. Following these consistent empirical findings, we expect that all arguments may lead to defensive reactions to a certain extent, but we predict that especially environmental arguments will meet these reactions that lower persuasion.

Thus, the main hypothesis is that, of the three types of arguments, environmental arguments will lead to the least persuasion, but only in recipients for whom the topic is of high personal relevance, that is, who eat relatively higher levels of meat. Animal welfare arguments may be somewhere in between: the outcomes may be seen as more direct and undeniable, and may lead to vivid mental images compared to environmental outcomes (de Boer and Aiking, 2022a). On the other hand, animal suffering may be more distant and less threatening than personal health-related suffering. Again, as these arguments can be compelling, they may still lead to defensive reactions to a certain extent because they may lead to “moral shock” that activates cognitive dissonance (Mathur et al., 2021a).

Some earlier studies made comparisons between these three types of arguments. For example, in one survey people were asked about their reasons to lower their meat consumption (Neff et al., 2018). The most reported reasons for reduction were financial costs and health, about 50% of the sample, while environment and animal welfare were reported as reasons by about 15% (for very similar findings, see Charlebois et al., 2016). A recent review of experimental studies on messages to lower meat consumption shows that very few compare the effects of health, environmental, and animal welfare arguments (Harguess et al., 2020). For example, one experimental study provided participants with one of the four types of “newspaper articles” on the environmental, animal, health, and personal identity consequences of meat consumption (Cordts et al., 2014). Animal welfare and health arguments had the strongest persuasive effects (on intention). The environmental consequences text led to the highest ratings of the article being unreliable, indicating defensiveness. Another experimental study, not included in the abovementioned review, provided participants with persuasive messages about environmental, animal, and health consequences, and disgust (Palomo-Vélez et al., 2018). The results showed that messages about disgust and animal welfare were more persuasive (on attitude) compared to messages about health and environment. Lastly, Wolstenholme et al. (2020) compared health and environmental arguments, and found that both types of arguments led to a significant decrease in meat consumption compared to a control group. To conclude, the available evidence is not unambiguous when it comes to what arguments are the most effective.

The present study aims to contribute to the literature in two ways. Firstly, the main outcome measure will not be attitudes or intentions but self-reported meat consumption. Secondly, as previously argued and found to be relevant in practice (Verain et al., 2017), we do not expect all people to react the same to the persuasive message. Therefore, we use theoretically and empirically based moderators, more specifically, indicators of personal relevance of the topic of persuasion (Petty and Cacioppo, 1986). Firstly, the topic can be experienced as more relevant when the level of meat consumption is higher (which will have more negative consequences). Secondly, the topic can be experienced as more relevant when the intention to reduce one's meat consumption is higher (more interest in the information). In both cases, the persuasive information will be processed more centrally, which may lead people to notice the (low) quality of the arguments.

The present study comprises an online exploratory experiment in which participants are asked to read a persuasive text about the environment-, the animal-, or the health-related outcomes of meat consumption, or a bogus text. The dependent variables are self-reported consumption of red meat and processed meat after 2 weeks. Participants' pre-test meat consumption and pre-test intention to lower their meat intake are assessed as potential moderators.

## Methods

### Recruitment

The recruitment ran from August to November 2019 in Italy. Calls to participate in an online study on meat consumption were published on Facebook. Particularly, the calls were posted on different types of Italian Facebook groups, like sport pages, groups related to beauty, diet, nature, or animal topics, and also several groups of University students of different faculties, all over Italy. This kind of data collection allowed us to obtain information from people of different ages, cultures, experiential backgrounds, and various interests. The call stated that a study was being conducted on the quality of information about meat consumption and about the topic of the environment, animals, and one's own health, and that participants could win one of three amounts of €50 in a lottery. By clicking on the link, respondents were routed to the online experiment system (Qualtrics).

### Design

This study comprised an online experiment with four independent conditions, three conditions with persuasive information and one control condition. The two individual difference moderators were assessed in the pre-test. The 2-week follow-up measurement concerned a self-report of red meat and processed meat consumption. Because the presently used experimental stimuli had not been used before, we aimed to include 50 participants per condition based on our experience with online experiments on persuasion. The study was approved by the Ethical Committee Psychology (ECP) of the faculty of Behavioural and Social Sciences of the University of Groningen (PSY-11819-S-0222).

### Procedure

After clicking the link, people who were interested arrived at the study's information and informed consent page. They were informed that they would be asked to read a text and answer some questions, and that they would be sent a link to a follow-up questionnaire after 2 weeks. Next, they entered the pre-test, with questions about demographics and psychological measurements. Then, they were randomized to one of the conditions, and asked to read the text. After that, they entered the immediate post-test with several psychological questions, and they were reminded of the upcoming follow-up test. After 2 weeks, they were sent automatically an email with a link to the self-report questionnaire.

### Measurements

Demographics were assessed: gender, age, and educational level of education, which was categorized as low/medium vs. high. Before asking participants about their meat consumption, it was explained in 101 words and six pictures what was meant by red meat and processed meat in the next questions.

Meat consumption at pre-test was assessed with a simplified version of the main outcome measure with two frequency questions: “On how many days of the week do you eat red meat?,” and “On how many days of the week do you eat processed meat?.” Eight answering options were provided, ranging from “1 day per week” (1) to “7 days per week” (7), and “never” (0). The mean score of the items was computed as the pre-test meat consumption score [*r*_(137)_ = 0.31, *p* < 0.001].

The intention to lower meat consumption was assessed with two items: “Are you planning to decrease your red meat/processed meat consumption in the next 2 months?” that could be answered on a five-point scale from “not planning at all” (1) to “strongly planning” (5). The items correlated significantly [*r*_(137)_ = 0.74, *p* < 0.001], and the average of the item scores was taken as the pre-test intention score.

Several other brief scales were applied in the pre-test and immediate post-test, assessing constructs that are not relevant to the present research question. Therefore, they will not be further presented here.

The follow-up measurement of meat consumption, firstly, assessed by self-report the number of days per week (“since the first study part about 2 weeks ago”) the participants had eaten meat during breakfast, lunch, as a snack, or at diner. This was asked for red meat and processed meat separately. Before asking participants about their portions, four pictures (two for red meat and two for processed meat) with different portions, labeled with grams, were depicted to support the estimation of portions in the self-report. Next, it was assessed how much meat they ate on each occasion (the portion), with the response options “50 g” (1), “100 g” (2), “150 g” (3), “200 g” (4), “250 g” (5), “300 g” (6), “more than 300 g” (7), or “never at this occasion” (0). For red meat and processed meat separately, the numbers of days of occasions were summed and multiplied with the recoded and summed portion scores. This led to a red meat and processed meat score for each participant to be used as the main dependent variables.

### Manipulation

Each of the three types of arguments was worded in a separate text, to be presented in one condition. The information was designed to be persuasive: it argued one-sidedly only on the negative effects.

The environmental conditions presented a text about the negative environmental outcomes of meat consumption, for example, “Eating red meat and processed meat is highly damaging to the environment. The meat industry contributes significantly to greenhouse gas emissions, deforestation, and climate change.” The text consisted of 205 words with information on how the meat industry contributes to air, water, and soil pollution. Factual information was given, for example, on the greenhouse effects of methane, on how much water 450 g of meat costs, and on the proportion of cereals taken by the meat industry. The text included eight pictures; three about air pollution, two about water pollution by stables, and three on soil pollution.

The animal conditions presented a text on the negative outcomes of meat consumption for animal living and well-being, for example, “the meat sector is one of the cruelest industries in the world. It has been scientifically proven that animals are sentient beings, capable of experiencing emotions such as fear, anxiety, and suffering.” The text consisted of 222 words with information on, for example, how animals are held in narrow and over-crowded stables, how they are subjected to ruthless practices (e.g., cutting tails), and how they are killed to be processed to meat. The text included eight pictures; six of cows and pigs in stables and two of rows of hanging slaughtered pigs in an abattoir.

The health conditions presented a text on the negative health outcomes of meat consumption, for example, “Reducing the consumption of red meat helps to preserve your health. The consumption of red meat and processed meat is associated with the risk of developing numerous diseases such as diabetes, hypertension, obesity, and cancer.” The text consisted of 199 words with information on, for example, the effects of meat consumption on blood cholesterol, on the carcinogenic effect of preparing meat, and on drug residues that meat can contain. The text included eight pictures; five with surgery scenes and three with close-ups of biological cells.

The control conditions presented a text on the production of mustard: “The production and consumption of mustard have many ancient origins: initially mustard was considered a plant with medicinal properties rather than an ingredient to be used in cooking.” The text consisted of 331 words with information on, for example, its history, how it is grown, and what it is used for. The text included three pictures of the mustard plant and seeds.

Each outcome text was complimented with a text designed to influence other possible determinants of meat consumption. This text was the same for all four conditions and consisted of 198 words. The social norm was addressed by briefly stating that more and more people lower their meat consumption. Response efficacy (or goal setting) was addressed by briefly stating that also lowering and not necessarily quitting meat consumption has beneficial effects. Self-efficacy was addressed by explaining that small changes add up, and 10 options were given to replace meat with “tasty, protein rich” products, with four pictures of such meals.

## Results

### Selection and Attrition Analyses

Of the 538 people who entered the online system, gave consent, and answered the first question, data from nine people were removed because of double or triple IP addresses. Of the remaining 529 participants, 180 (34%) had complete self-report data on their consumption at the post-test after 2 weeks. To select for sufficient exposure and serious engagement, only participants who were randomized to one of the four conditions, and who were present in the online system for over 5 min were included, leaving 137 participants. These selected 137 participants were compared to the 392 who were not selected on pre-test meat consumption, pre-test intention, and age using analysis of variance (ANOVA), and on gender and education type using Chi-square tests. These analyses revealed no significant differences on all five variables (all *ps* > 0.22).

### Participant Characteristics

Of the 137 participants included, 24% were men, their level of education was mostly high (84% had a higher preliminary training or a bachelor's or master's level), and the mean age was 28.4 [standard deviation (SD) = 11.3]. The mean score on pre-test meat consumption, the number of days participants eat meat was 1.78 (SD = 1.12): with regard to red meat, 84% ate it <3 days per week; with regard to processed meat, this percentage was 73%. The mean score for the intention to decrease one's meat consumption was 2.48 (SD = 1.34).

### Randomization and Manipulation Check

The 137 participants were randomized to the four conditions but due to selective drop-out the number differed somewhat: The environment condition *n* = 38; the animal condition *n* = 23; the health condition *n* = 35; and the control condition *n* = 41. To test whether participants in the conditions still had similar characteristics, the conditions were compared on the same five variable as above. All test were not significant (*p*s > 0.28), suggesting that randomization was successful. After exposing participants to the manipulation, they were asked how reliable they found the information to be. The mean score was 5.10 (SD = 1.30), and the conditions did not differ significantly (*p* > 0.68).

### Preparatory Analyses

Both dependent variables, red meat consumption and processed meat consumption, did not meet the assumption of normality; both were skewed to the left, showing relatively more people who ate no or little meat, with skewness and kurtosis beyond 1 and −1. Therefore, the raw scores were recoded into quintiles, leading to five increasing levels of consumption with each having a similar number of participants. The correlation between the red and processed meat score was *r*_(137)_ = 0.47, *p* < 0.001. Both variables now met the requirements of the ANOVA.

To start with, the main effect of condition on post-test red meat consumption and processed meat consumption was tested in an analysis of covariance (ANCOVA), with pre-test meat consumption and pre-test intention as covariates. For both dependent variables, the main effect of condition was not significant: for red meat consumption, *F*_(3, 134)_ = 1.03, *p* = 0.38, η^2^ = 0.023, for processed meat consumption, *F*_(3, 134)_ = 1.27, *p* = 0.29, η^2^ = 0.028. None of the contrasts were significant (all *ps* > 0.11).

### Interaction Effects

Next, the moderation effects of pre-test meat consumption and pre-test intention were studied. Because both variables were significantly correlated, *r*_(137)_ = −0.30, *p* < 0.001, a confounding analysis was conducted: Both interactions, condition × pre-test meat consumption, and condition × pre-test intention were tested in a single saturated model (Yzerbyt et al., 2004), one for red meat consumption and one for processed meat consumption.

In the model with red meat consumption as the dependent variable, the interaction between pre-test meat consumption and condition was significant, *F*_(3, 125)_ = 3.08, *p* = 0.03, η^2^ = 0.07, while the interaction between pre-test intention and condition approached significance, *F*_(3, 125)_ = 2.27, *p* = 0.084, η^2^ = 0.052. In the model with processed meat consumption as the dependent variable, both interactions were not significant (*p* > 0.22), and the model without these interactions showed no significant main effect of condition (*p* > 0.20).

### Pre-Test Meat Consumption as a Moderator

**Figure 1:**
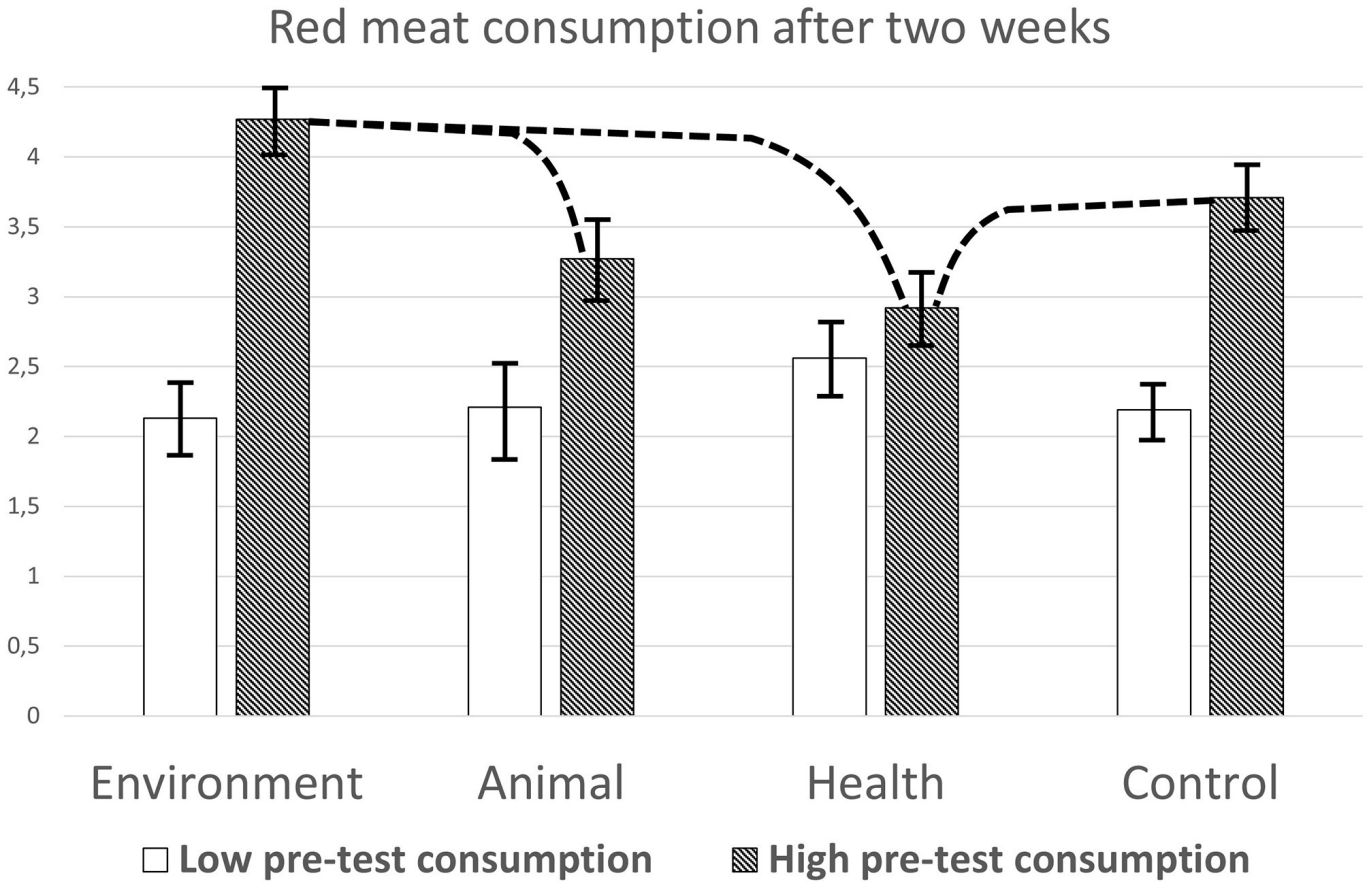
Levels of self-reported red meat consumption after 2 weeks in the four conditions, according to the pre-test level of meat consumption. Dotted lines represent significant contrasts. Error bars refer to standard errors (SEs).

To further understand the moderation effects on red meat consumption, firstly, the effects of condition in participants with high and low pre-test consumption were computed. Therefore, the complete data set (*n* = 137) was modeled to represent two levels of pre-test meat consumption, by subtracting and adding one to the individual standardized scores (*z*-scores), respectively (Siero et al., 2009). The estimated means on the categorized measure of post-test red meat consumption are depicted in Figure 1.

When pre-test consumption was modeled as low, the main effect of condition was not significant, *F*_(3, 125)_ = 0.48, *p* = 0.70, η^2^ = 0.011. None of the contrasts between the conditions was significant (all *ps* > 0.27).

When pre-test consumption was modeled as high, the main effect of condition was significant, *F*_(3, 125)_ = 4.10, *p* = 0.008, η^2^ = 0.09. Contrast analyses showed that red meat consumption in the health condition (*M* = 2.92) was significantly lower than in the environment condition (*M* = 4.27, *p* = 0.001, 95% confidence interval (CI) difference −2.12 to −0.54), and the control condition (*M* = 3.71, *p* = 0.03, 95% CI difference −1.46 to −0.08). In addition, red meat consumption in the animal condition (*M* = 3.27) was significantly lower compared to the environment condition (*M* = 4.27, *p* = 0.029, 95% CI difference −1.91 to −0.10). Consumption in the control condition was also lower than in the environment condition, although the difference only approached significance (*p* = 0.099).

Next, correlations between pre- and post-test meat consumption, controlling for pre-test intention, were computed within each of the four conditions. In the environment and control condition, the correlations were significant, *r*_(35)_ = 0.67, *p* < 0.001, 95% CI bootstrap 0.46–0.83 and *r*_(38)_ = 0.71, *p* < 0.001, 95% CI bootstrap 0.51–0.85. In the animal condition, the correlation approached significance, *r*_(20)_ = 0.41, *p* = 0.056, while in the health condition, the correlation was not significant, *r*_(32)_ = 0.15, *p* = 0.42.

### Pre-Test Intention as a Moderator

**Figure 2:**
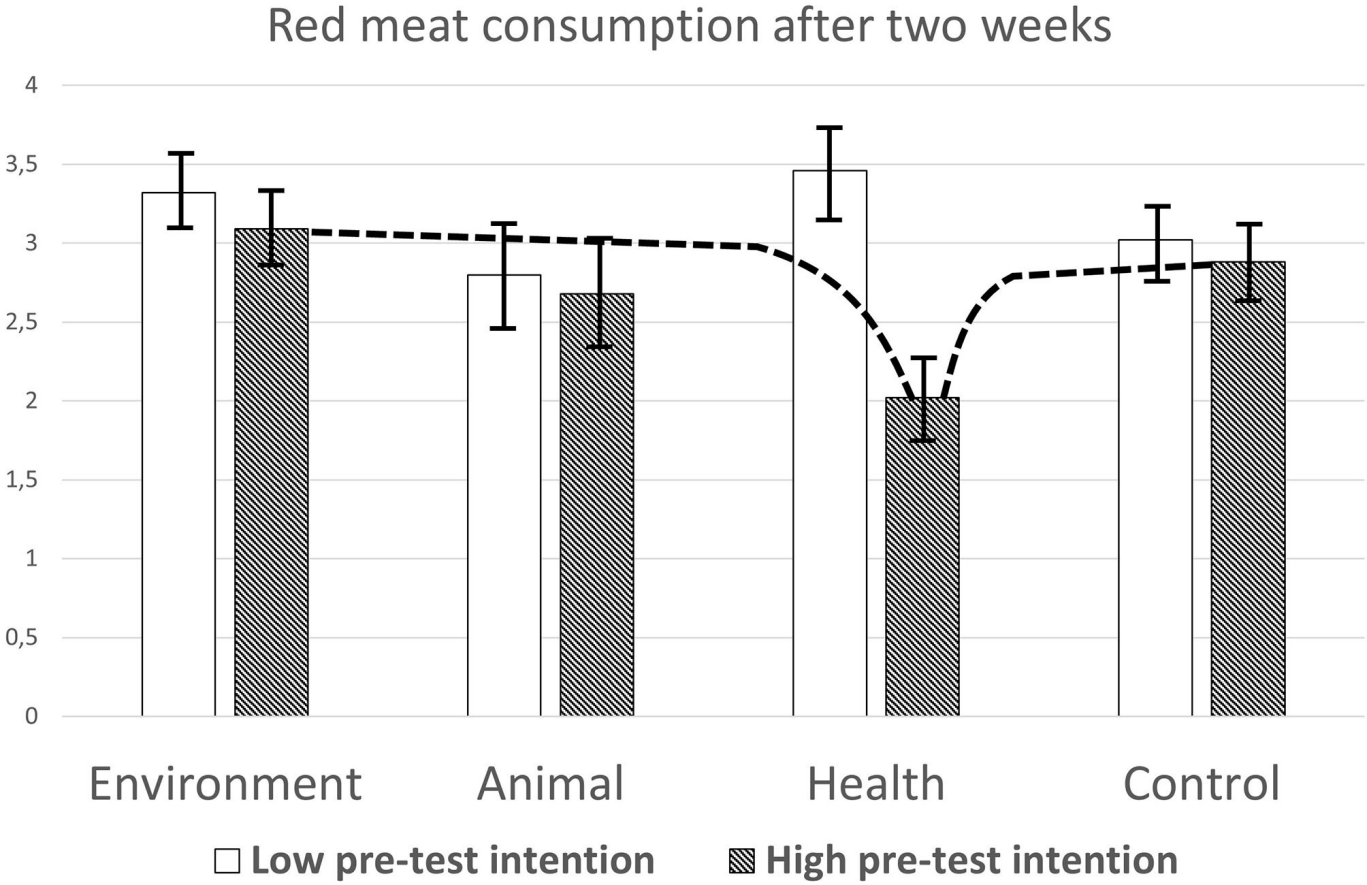
Levels of self-reported red meat consumption after 2 weeks in the four conditions, according to the pre-test intention level. Dotted lines represent significant contrasts. Error bars refer to SEs.

To further understand the effects of moderation on red meat consumption, firstly, the effects of the condition in participants with high and low pre-test intention were computed, using the same procedure as mentioned above. The estimated means on the categorized measure of post-test red meat consumption are depicted in Figure 2.

When pre-test intention was modeled as low, the main effect of condition was not significant, *F*_(3, 125)_ = 1.07, *p* = 0.37, η^2^ = 0.025. None of the contrasts between the conditions were significant (all *ps* > 0.20). When pre-test intention was modeled as high, the main effect of condition was significant, *F*_(3, 125)_ = 2.80, *p* = 0.043, η^2^ = 0.063. Contrast analyses showed that red meat consumption in the health condition (*M* = 2.02) was significantly lower than that in the environment condition (*M* = 3.09; *p* = 0.006, 95% CI difference −1.81 to −0.32) and in the control condition (*M* = 2.88, *p* = 0.025, 95% CI difference −1.6 to −0.11).

Next, the correlations between pre-test intention and post-test red meat consumption, controlled for pre-test meat consumption, in each of the four conditions were computed. The correlation in the health condition was significant, *r*_(32)_ =-0.46, *p* = 0.007, 95% CI bootstrap −0.67 to −0.21. In the other three conditions, the correlations were also negative but not significant, and smaller than −0.14.

## Discussion

This study aimed to explore the persuasiveness of the three types of arguments to reduce meat consumption. The findings are only partly according to our expectations: one observation is that the persuasive messages only influenced red meat consumption but not processed meat consumption. It may be that people do not approach their consumption of red meat and processed meat in the same way (Pfeiler and Egloff, 2018), similar to the finding that people differentiate between fruit and vegetable consumption (Chapman and Armitage, 2012; Elbert et al., 2016). In addition, personal relevance operationalized as pre-test meat consumption and pre-test intention moderated the effects of persuasive messages. All statistical analyses for one moderator were controlled for possible effects of the other moderator, suggesting independent, non-overlapping effects of both moderators.

In participants with relatively high pre-test meat consumption, health arguments led to lower red meat consumption, compared to environmental arguments and to the control condition. Especially, the comparison with the control condition shows that health arguments were effective; they lowered red meat consumption compared to when people received no arguments. In the environmental argument condition, red meat consumption was higher compared to the control condition (although the test only approached significance) and to the animal welfare condition. The data do not show whether environmental arguments actively increased red meat consumption; they might have been inert, with little persuasive power, or they might activate negative thoughts that inhibited persuasion because they were perceived as weak. The finding that environmental arguments are not always effective in the general population is in line with earlier findings (de Boer et al., 2013). They propose to develop messages that address multiple values (Lai et al., 2020; Wolstenholme et al., 2020). However, the environmental and animal welfare consequences of meat consumption are complex and can be formulated in different ways to motivate people (Giacoman et al., 2021; de Boer and Aiking, 2022a). Therefore, it is too early to formulate a definitive conclusion about the effectiveness of different types of arguments. In summary, in this study, health arguments were the most effective, and environmental arguments the least effective. Animal arguments fell somewhere in between. In participants with relatively low pre-test meat consumption, there were no significant differences in red meat consumption post-test. This segment already consumed little or no meat.

Another way to understand the moderation effect of pre-test meat consumption is to look at the correlations within conditions. As illustrated in Figure 1, the relationships between the pre-test meat consumption and post-test red meat consumption were positive and significant in the environmental and control conditions. Thus, behavior predicted behavior, and this was not changed by the persuasive messages. However, in the health argument condition, this correlation was no longer significant. Health arguments seemed to have disrupted the relationship, leading to a change in red meat consumption that was not in line with earlier behavior. It might be argued that only health arguments renewed people's decision-making regarding red meat consumption.

The moderating effects of pre-test intention seemed somewhat less pronounced and a little different: in participants with relatively high pre-test intention, environmental arguments did not influence red meat consumption compared to the control condition, while health arguments led to lower post-test red meat consumption compared to both environmental and control conditions. In participants with relatively low pre-test intention, the arguments made no difference at all. This segment may consist of people who do not want to change their meat consumption because they have a positive attitude toward meat or because they have already lowered their meat consumption and are not willing to change it further. In the development of interventions, such a segmentation of the meat consumer population is essential (Knaapila et al., 2022). Looking at the relationships within conditions, as illustrated in Figure 2, only in the health argument condition pre-test intention was related to post-test red meat consumption. This suggests that in the other conditions people's intentions were not relevant to the persuasive effect; only when people read health arguments, their initial intentions were used to guide their behavior. The effects of both moderators are in line with the notion that only health arguments influenced the decision process regarding red meat consumption beyond the established, possibly habitual, earlier behavior.

One of the limitations regarding the persuasive texts is that they are confounded: the environmental consequences that are presented refer to higher-level, more abstract, or distant outcomes, while the health consequences that are presented concern the concrete and proximal physical body. The environmental consequences might also have been brought closer to the individual (e.g., consequences for one's personal safety), while the health consequences might have referred to more abstract and distal health effects (e.g., health effects on a population level, epidemiological, and financial). It cannot be ruled out that other formulations referring to the different levels of outcomes regarding the environment and health (and animal welfare) may have different effects.

Furthermore, this study tested the three arguments separately. Although this is how arguments and persuasive messages are often used in practice, combining them into an integrated message might be more powerful, as different recipients may have different values and need different arguments. However, the findings of combining arguments are mixed (Mathur et al., 2021b) but promising (Lai et al., 2020; Wolstenholme et al., 2020).

In addition, the persuasive texts used strong language, with phrases such as: highly damaging to the environment, the cruelest industry, and numerous diseases. Although the information presented was factual, this framing may have contributed to the “moral shock” that activated cognitive dissonance (Mathur et al., 2021a) or defensive self-regulatory reactions. Therefore, it is important not only to distinguish between types of arguments but also to distinguish between formulations of arguments.

Another limitation is related to the level of pre-test meat consumption in the sample. Around 84 and 73% of the participants ate red or processed meat, respectively, not even 3 days per week. In the statistical method that tested the effects at different levels of the pre-test red meat consumption moderator, the “low” level referred to an average red meat consumption frequency of <0.5 days per week, while the “high” level referred to an average red meat consumption frequency of almost 3 days per week. In relation to the current Italian red meat consumption, the “high” group might probably be better classified as average (Farchi et al., 2017). Moreover, these low levels of meat consumption may partly reflect the self-selected sample that consisted predominantly of young women with a high level of education. This selection may be related to the topic of meat reduction but also to involvement in (one of) the three types of arguments that were mentioned in the recruitment text. In addition, women seem to differ in their perceptions of meat consumption and react differently to information about meat (Dowsett et al., 2018).

Based on their level of meat consumption, the self-selected sample can be classified as consisting of flexiterians or meat reducers: meat consumers who deliberately consume less meat, for various reasons (Dagevos, 2021; Knaapila et al., 2022). In a representative Australian sample, about 20% of participants identified themselves as meat reducers (Malek and Umberger, 2021), but different studies report different percentages (Dagevos, 2021). When the present sample would be classified as flexitarian, the generalizability of the findings may be limited to this segment: Knaapila et al. (2022) conclude that this “middle segment” might be the most responsive to interventions aimed at lowering meat (see also Verain et al., 2022). Future studies will have to unravel the persuasive power of different arguments in different segments.

One issue that must be considered in interpreting the present results is the Italian food culture. Food cultures differ in meat consumption patterns and other dietary contents (de Boer and Aiking, 2018). In addition, people from different cultures may differ in their opinions about what sustainability actions should be taken. For example, people from Eastern and Southern European countries (including Italy) were less inclined to endorse the idea that consumers have a role to play in making their food system sustainable (de Boer and Aiking, 2022b). Furthermore, the Italian food culture may have specific characteristics that influence psychological determinants related to meat consumption. For example, Wolstenholme et al. (2021) suggest that because the Italian diet is predominantly plant based, people have more opportunities to lower their meat consumption, making is easier. This implies that in Italy lowering meat consumption would be more an issue of motivation than of control. As the experimental conditions in the present experiment are largely motivational (i.e., providing arguments/information about consequences), in cultures in which there are fewer opportunities to omit or replace meat the results may be different.

In addition, the pre-test meat consumption measure simply assessed the number of days participants ate meat, red or processed, while the post-test consumption was the full assessment of number of days, portion sizes, and differentiated between red and processed meat. Yet, in the control condition without interference from the arguments on meat consumption, the correlation between these measures was significant, positive, and substantial, 0.71. Thus, despite its simplicity, the pre-test measure was a reasonably good predictor of meat consumption after 2 weeks.

Lastly, the relatively low number of participants in the conditions may have undermined the statistical power of the study. However, the effect sizes of the main effects at high levels of the moderators were moderate, while the values of *p* for the crucial comparison between the environmental and health message were <0.01. These effects seem robust, despite the smaller number of participants. In addition, the specific moderation analysis used did not further split the sample but used the complete data set. Still, it cannot be ruled out that the positive predictive value of the analyses is compromised, meaning that we have to be careful even to believe the significant effects (Button et al., 2013). This analysis on the statistical power implies that a study with more participants is needed and will be conducted to replicate the present findings.

This exploratory study shows that, although arguments may refer to objective and compelling outcomes, they are not always perceived as such and used as reasons to change one's behavior. This seems to be the case with environmental arguments that are true but may seem far-fetched to many people. Although the facts about the environmental outcomes of meat consumption may come from experts, they may still be perceived as weak arguments (Mizrahi, 2013). Animal welfare outcomes may not have been perceived as far-fetched, but they may rely on empathic motivation or ability toward other living creatures. On the other hand, people seemed to be convinced by health arguments that presented highly probabilistic outcomes but very close to the individual recipient. This study did not unravel exactly why different arguments have different effects, but it does show that they do differ. Our finding may inspire further studies on the three core arguments, and it may support the practice of persuasion to carefully test specific arguments, in isolation or in combination in target populations.

## Data Availability Statement

The raw data supporting the conclusions of this article will be made available by the authors, without undue reservation.

## Ethics Statement

The studies involving human participants were reviewed and approved by Ethical Committee Psychology (ECP) of the University of Groningen. The patients/participants provided their written informed consent to participate in this study.

## Author Contributions

AD and VR designed the study in collaboration. The execution of the study and the initial writing of the manuscript was done by VR. AD conducted the statistical analyses and edited the manuscript to its final version. Both authors contributed to the study design, execution, and writing of this manuscript.

## Funding

The University of Groningen, Department of Psychology, financed the study, funded the open access publication costs.

## Conflict of Interest

The authors declare that the research was conducted in the absence of any commercial or financial relationships that could be construed as a potential conflict of interest.

## Publisher's Note

All claims expressed in this article are solely those of the authors and do not necessarily represent those of their affiliated organizations, or those of the publisher, the editors and the reviewers. Any product that may be evaluated in this article, or claim that may be made by its manufacturer, is not guaranteed or endorsed by the publisher.
